# Electrochemical Engineering of Nanoporous Materials

**DOI:** 10.3390/nano8090691

**Published:** 2018-09-06

**Authors:** Abel Santos

**Affiliations:** 1 School of Chemical Engineering, The University of Adelaide, Adelaide, SA 5005, Australia; 2 Institute for Photonics and Advanced Sensing (IPAS), The University of Adelaide, Adelaide, SA 5005, Australia; 3 ARC Centre of Excellence for Nanoscale BioPhotonics (CNBP), The University of Adelaide, Adelaide, SA 5005, Australia

Nanoporous materials are outstanding platforms due to their unique chemical and physical properties at the nanoscale, which make them suitable candidates to develop advanced materials and systems for a plethora of applications, including catalysis and photocatalysis [1,2,3,4,5,6,7], energy harvesting and storage [8], photonics and optoelectronics [9,10], nanomedicine [11,12,13,14,15], and filtration and separation [16,17,18]. Among different methods, electrochemical fabrication techniques offer many advantages over conventional nanofabrication methods to produce nanoporous materials with precisely engineered properties, such as controllability, reproducibility, high resolution, scalability, high-throughput, cost-competitiveness, and time-efficient processes. Despite numerous advances in this area, electrochemical engineering of nanoporous materials remains a highly dynamic and broad research field that continues to enable excellent opportunities for further trans-disciplinary fundamental and applied research.

In this context, this Special Issue of *Nanomaterials* collates a series of illustrative examples on several fundamental aspects and inter-disciplinary applications of nanoporous materials produced by different electrochemical and chemical methods, from energy to drug delivery. It is thus expected that the field of electrochemically engineered nanoporous materials will continue to grow and spread towards more sophisticated applications. Metallic and semiconductor nanoporous materials can enable the precise control of light–matter interactions, such as surface plasmon resonance, photonic crystal and slow photon effect at the nanoscale for optical sensing and biosensing, energy harvesting, and environmental remediation applications. Nanoporous materials with well-defined nanostructures enable new opportunities to study molecular interactions to develop advanced materials with unique chemical and physical properties for ultra-efficient separation and filtration processes, such as water desalination. Nanoporous materials based on inert and non-cytotoxic materials provide an excellent matrix to load and accommodate therapeutics, which can be passively or actively released by local or remote triggers in a timely fashion for personalised medical therapies. These structures also provide unique template platforms for the synthesis of other nanostructures with controlled geometric features.

Finally, I would like to thank all authors of this Special Issue of *Nanomaterials* for their contributions and all referees for their valuable comments, suggestions and time, as well as the editorial office for their constant and swift support throughout. 
